# Resistance to temephos and deltamethrin in *Aedes aegypti* from Brazil between 1985 and 2017

**DOI:** 10.1590/0074-02760180544

**Published:** 2019-04-29

**Authors:** Denise Valle, Diogo Fernandes Bellinato, Priscila Fernandes Viana-Medeiros, José Bento Pereira Lima, Ademir de Jesus Martins

**Affiliations:** 1 Fundação Oswaldo Cruz-Fiocruz, Instituto Oswaldo Cruz, Laboratório de Biologia Molecular de Flavivírus, Rio de Janeiro, RJ, Brasil; 2 Fundação Oswaldo Cruz-Fiocruz, Instituto Oswaldo Cruz, Laboratório de Fisiologia e Controle de Artrópodes Vetores, Rio de Janeiro, RJ, Brasil; 3 Instituto de Biologia do Exército, Rio de Janeiro, RJ, Brasil; 4 Instituto Nacional de Ciência e Tecnologia em Entomologia Molecular, Rio de Janeiro, RJ, Brasil

**Keywords:** Aedes aegypti, Brazil, insecticide resistance, temephos, deltamethrin, vector control

## Abstract

**BACKGROUND:**

*Aedes aegypti* populations in Brazil have been subjected to insecticide selection pressures with variable levels and sources since 1967. Therefore, the Brazilian Ministry of Health (MoH) coordinated the activities of an *Ae. aegypti* insecticide resistance monitoring network (MoReNAa) from 1999 to 2012.

**OBJECTIVES:**

The objective of this study was to consolidate all information available from between 1985 and 2017 regarding the resistance status and mechanisms of Brazilian *Ae. aegypti* populations against the main insecticide compounds used at the national level, including the larvicide temephos (an organophosphate) and the adulticide deltamethrin (a pyrethroid).

**METHODS:**

Data were gathered from two sources: a bibliographic review of studies published from 1985 to 2017, and unpublished data produced by our team within the MoReNAa between 1998 and 2012. A total of 146 municipalities were included, many of which were evaluated several times, totalling 457 evaluations for temephos and 274 for deltamethrin. Insecticide resistance data from the five Brazilian regions were examined separately using annual records of both the MoH supply of insecticides to each state and the dengue incidence in each evaluated municipality.

**FINDINGS:**

*Ae. aegypti* resistance to temephos and deltamethrin, the main larvicide and adulticide, respectively, employed against mosquitoes in Brazil for a long time, was found to be widespread in the country, although with some regional variations. Comparisons between metabolic and target-site resistance mechanisms showed that one or another of these was the main component of pesticide resistance in each studied population.

**MAIN CONCLUSIONS:**

(i) A robust dataset on the assessments of the insecticide resistance of Brazilian *Ae. aegypti* populations performed since 1985 was made available through our study. (ii) Our findings call into question the efficacy of chemical control as the sole methodology of vector control. (iii) It is necessary to ensure that sustainable insecticide resistance monitoring is maintained as a key component of integrated vector management. (iv) Consideration of additional parameters, beyond the supply of insecticides distributed by the MoH or the diverse local dynamics of dengue incidence, is necessary to find consistent correlations with heterogeneous vector resistance profiles.

Several factors modulate the occurrence of dengue epidemics. The transmission of the dengue virus is related to the density and susceptibility to the virus of the human population, along with the density and dispersion of its vector, *Aedes aegypti*, a mosquito that is extremely well-adapted to environments modified by humans.^1^ ^,^ ^2^ In Brazil, the distribution of dengue epidemics is very heterogeneous due to: the territorial dimension, climatic diversity, and large number of densely populated urban centres in the country; the occurrence of water supply failures, irregular waste collection, problems with sanitation, and construction and repair work on urban roads often paralysed leading to the accumulation of water; behavioural issues as divergences in the identification of ‘public’ and ‘private’ spaces, among others.^3^ Since an epidemic occurred in Rio de Janeiro in 1986, dengue fever has been a relevant public health problem in the country. Brazil is hyperendemic for this arbovirus, since the four serotypes, dengue virus type 1 (DENV-1) (since 1986), DENV-2 (1990), DENV-3 (2001), and DENV-4 (2010), circulate periodically in the country’s populace.^4^ ^,^ ^5^ ^,^ ^6^ ^,^ ^7^ Moreover, due to the seasonality of the vector’s populations, different regions of the country face different periods of dengue epidemic in their hot and rainy seasons.^3^ ^,^ ^8^

Since specific drugs and effective vaccines against the dengue virus are not available for large-scale application, dengue control and prevention depend mainly on actions directed towards its vector. Currently, there is growing recognition that dengue control solutions surpass the health sector and are dependent on structural actions, such as sanitation, garbage collection, and water supply maintenance, as well as on community mobilisation and participation in the elimination of potential breeding sites, many of which are located indoors. In spite of this, in many cases public managers and even citizens tend to confuse vector control with vector chemical control, and the consequence of this is the intensified use of insecticides, which culminates in the dissemination of insecticide resistance in *Ae. aegypti* populations.^9^ ^,^ ^10^ ^,^ ^11^ ^,^ ^12^ ^,^ ^13^ ^,^ ^14^

In Brazil, dengue vector populations have been subjected to selection pressures by different classes of insecticides. Since 1967, the continued use of organophosphate (OP) pesticides has selected for resistant individuals. For a long time, only the OP temephos was approved by the World Health Organization (WHO) for use in drinking water, and therefore in the control of *Ae. aegypti* larvae.^15^ The first reports of alterations in the susceptibility status of *Ae. aegypti* to temephos appeared by the end of the 1990s.^16^ As a result, in 1999 the Brazilian Ministry of Health (MoH), acting through the National Dengue Control Program (PNCD), coordinated the creation of the National Network for Monitoring the *Aedes aegypti* Resistance to Insecticides (MoReNAa), a partnership of the government with several research laboratories that was active until 2012. This network’s aim was to monitor the resistance status of the vector to the main insecticide compounds employed at the national level in municipalities representative of the dengue situation in the country as a whole.^11^ ^,^ ^17^

As a result of both the confirmation of the development of temephos resistance and the approval of new larvicides for use in drinking water, Brazil adopted the use of other compounds against larvae in addition to the OP temephos, namely *Bacillus thuringiensis* var. *israelensis* (*Bti*) and insect growth regulators (IGRs).^18^ In addition, in 2006 the criterion for when temephos substitution with an alternative insecticide should be enacted was modified: the exchange was recommended when the RR_95_ (resistance ratio) of vector populations reached values above 3.0, instead of 10.0 as previously required. The reason for this change was the long time period required to organize the distribution of any new product throughout the country. The objective in this case was to preserve the insecticide, suspending its use before resistance to it became established, a situation that would definitely compromise its use in the field.^19^ ^,^ ^20^ ^,^ ^21^ For academic purposes, this same criterion has also been used to classify the resistance status of vector populations to deltamethrin.^13^ ^,^ ^22^

Regarding adult vectors, resistance to pyrethroid (PY) pesticides was first observed in 2000, shortly after the beginning of their application in the field.^23^ However, until 2009, with the exception of São Paulo state, the PY deltamethrin was employed throughout the country in spatial and residual applications for the control of *Ae. aegypti*. The detection of PY resistance dissemination in several localities^24^ ^,^ ^25^ resulted in its substitution with the OP malathion.^26^ However, in addition to the domestic use of PY, which is available on the retail market, this class of insecticides is used in the control of vectors of Chagas disease, malaria, and leishmaniasis in several Brazilian regions. It should be noted that in Brazil, only compounds and formulations approved by the WHO are used for such purposes, in the interests of protecting public health.^18^ ^,^ ^27^

In this study, we presented a systematic review of the resistance statuses of Brazilian *Ae. aegypti* populations against temephos and deltamethrin, the two main compounds employed at the national level in the control of the larvae and adults, respectively, of this dengue vector. This review was based on two data sources: results presented by our team to the PNCD within the scope of the MoReNAa, but still unpublished, and a bibliographic review of the published results of other studies. Results of the studies reviewed consisted of those obtained from: (1) qualitative bioassays for the detection of adult resistance to deltamethrin, using only one diagnostic dose of the insecticide with two different methodologies; (2) quantitative bioassays of larvae with temephos and of adults with deltamethrin, based on the utilization of several insecticide doses; and (3) quantification of the activities of the main enzymatic classes related to metabolic resistance, including mixed-function oxidase (MFO), esterase (EST), and glutathione S-transferase (GST) enzymes, and also one related to the OP target site, acetylcholinesterase (ACE). Regarding the PY target site, the voltage-gated sodium channel (*Na*_*V*_ ), we also examined published molecular data on the frequencies of resistant alleles of *kdr* markers at positions 1016 and 1534 in the target site. In the bibliographic review performed, we confirmed that much of the available data were concentrated in two previous studies: (1) a review by Ranson et al.^28^ of the global insecticide resistance status of dengue vectors to OPs and PYs; and (2) Moyes et al.^29^, who updated and expanded the previous study in the context of the Worldwide Insecticide Resistance Initiative (WIN) supported by the WHO, while also mapping the global geographic distribution of resistance and the associated mechanisms thereof. It is noteworthy that both studies recognised Brazil as one of the few countries with a well-structured insecticide resistance monitoring program focused on *Ae. aegypti*.

In all cases, for each of the five Brazilian regions we sought to correlate the data for two annual records: (1) the MoH supply of insecticides provided to each state, and (2) the dengue incidence in each evaluated municipality. As an outcome of this effort, we consolidated and updated the information available on the temephos and deltamethrin resistance of Brazilian *Ae. aegypti* populations published until July 2017. This was done to advance our understanding of the pesticide resistance dynamics and their associated mechanisms in *Ae. aegypti* populations.

## MATERIALS AND METHODS

*Mosquitoes* - Samples were collected in the field between 2005 and 2011. *Ae. aegypti* eggs were collected with ovitraps following MoReNAa recommendations.^20^ ^,^ ^30^ ^,^ ^31^ In the laboratory, 1,000 larvae were raised per plastic basin (27 × 19 × 7 cm) containing 1L of partially dechlorinated water at 26 ± 1ºC. Every three days, 0.5 g of cat food (Friskies®, Purina, São Paulo, SP) was added to the basin as food for the larvae.^13^ Pupae were transferred to cylindrical cardboard cages (18 × 17 cm), and the identification and sorting of the *Ae. aegypti* adults that emerged from them was done according to Consoli and Lourenço-de-Oliveira.^32^ Guinea pigs, anesthetised with ketamine and xylazine,^33^ were used as a blood meal source for the adults once a week for four weeks. Whenever possible, F1 or F2 mosquitoes were employed in the assays performed.

*Insecticides* - Data on the temephos and deltamethrin sources employed in the assays performed are presented in Supplementary data (Table I).

*Quantitative bioassays* - Temephos dose-response bioassays with larvae were performed following WHO^34^ procedures, a protocol that was also described by Lima et al.^30^ and Braga et al.^31^. For each municipality, at least three trials were carried out on different days, totalling at least 2,400 exposed larvae. Each trial consisted of 10 different temephos concentrations, four replicates per concentration, and 20 larvae per replicate. Mortality was recorded after 24 hours.

Starting in 2009, adults of some vector populations were also submitted to deltamethrin dose-response bioassays, using an adapted version of the impregnated paper methodology.^13^ ^,^ ^35^ ^,^ ^36^ ^,^ ^37^ Each replicate employed 15-20 females, which were one to five days old and not fed blood. Three replicates were used per concentration, and between eight and 10 concentrations were tested per assay. At least three trials were performed for each population on different days, totalling 1,080 to 1,800 females. Mosquitoes were exposed to the insecticide for one hour, and then transferred to recovery tubes, without insecticide, and any mortality was recorded 24 h later.

In all cases, the susceptibility reference lineage Rockefeller^38^ was used to calibrate the assays and to calculate RR_95_ values.

*Qualitative bioassays* - In many cases, adult resistance to deltamethrin was investigated with diagnostic-dose (DD) assays using either insecticide-impregnated bottles or papers, based on the methodologies of the Centers for Disease Control and Prevention (CDC) and WHO, respectively.^35^ ^,^ ^39^ In both bioassays, replicates of 15-20 females, one to five days old and not fed blood, were used. For each vector population, at least three trials were performed on different days.

For the CDC methodology, a control bottle impregnated with solvent alone (acetone) and three bottles impregnated with 5 μg of deltamethrin were used per test. According to this methodology, the DD is defined as the lowest deltamethrin concentration that kills 100% of Rockefeller mosquitoes in 30 min. The assay took 30-120 min, and knockdown records were taken every 15 min. Females were then transferred to recovery cardboard cages, and mortality counts were made 24 h later.^23^ ^,^ ^39^ ^,^ ^40^

For the WHO methodology,^35^ each assay consisted of three replicates with papers impregnated with 3.65 mg deltamethrin/m^2^ (0.009%) and one control tube containing paper impregnated only with silicone oil.^36^ In the WHO methodology, the DD is equivalent to twice the lethal concentration that kills 99% (LC_99_) of Rockefeller mosquitoes. After being in contact with the insecticide for 1 h, the mosquitoes were transferred to recovery tubes, and their percent mortality was determined 24 h later.

*Biochemical tests* - Enzymes related to metabolic resistance were investigated in individual specimens following the protocols described by Valle et al.^41^ and Montella et al.^20^ for adults, and by Viana-Medeiros^42^ for larvae. For each population, approximately 90 larvae (L3 final or L4 initial) or 90 adult females (24 h old and not fed blood) were used. The activities of MFO, EST, and GST enzymes were then quantified. For ESTs, three different substrates were used: alpha-naphthyl (α-EST), beta-naphthyl (β-EST), or p-nitrophenyl (ρNPA-EST) acetate. The activity of the OP target, ACE, was also measured; in this case, in addition to its inhibition by propoxur (AChI), the ACE total activity (AChE) was also quantified.

*Data analyses* - The following criteria proposed by David and Zahar^43^ were adopted in the present study to interpret the results of qualitative bioassays: less than 80% mortality indicates resistance, mortality above 98% indicates susceptibility, and intermediate values point to incipient resistance.

Cohen’s kappa coefficient^44^ ^,^ ^45^ was used to compare the results of CDC and WHO adult qualitative assays. This coefficient (κ) is a proxy for data agreement, which varies between zero and one. Two mortality cut-off points were used in these comparisons, 80%^43^ and 90%^46^.

In the case of dose-response assays, lethal concentrations (LC) were calculated with Polo-PC software^47^ via probit analysis^48^ and used to calculate RR_95_ values through comparisons with Rockefeller data. For both temephos and deltamethrin, the functional criterion adopted by the MoReNAa in 2006 was used,^11^ ^,^ ^20^ which states that a RR_95_ value above 3.0 is indicative of resistance.

Results of biochemical assays were classified following Valle et al.^41^ and Montella et al.,^20^ using the Rockefeller 99th percentile obtained for each enzyme as a cut-off point. For each population and class of enzymes, the activity was then classified as unchanged, altered, or highly altered by the insecticide if the percentage of the samples above the Rockefeller 99th percentile was below 15%, between 15% and 50%, or above 50%, respectively. These percent activity change results for EST, MFO, and GST enzymes were also used to calculate the mean percent change in metabolic resistance (‘X_Metab_Resist’), a secondary measure corresponding to the mean of the changes in EST, MFO, and GST activities in each population. This latter measure was used to compare metabolic and target-site resistance mechanisms, as described in detail in the ‘Results’ section. AChE activity was evaluated as described above, through comparisons with the Rockefeller 99th percentile. Additionally, AChI assays were classified according to WHO criteria, which state that activity inhibition above 70% indicates unaltered ACE activity.^49^

*Delivery of insecticides used in public health* - In Brazil, the MoH is responsible for distributing the insecticides used to control important disease vectors with relevance to public health to all states.^13^ The annual delivery records of insecticides used against *Ae. aegypti* from 2003 to 2014 are presented herein. The larvicides employed were temephos, *Bti*, and IGRs (the chitin synthesis inhibitors novaluron and diflubenzuron, and the juvenile hormone analogue pyriproxifen). The adulticides employed were malathion (an OP) and several PY insecticides (deltamethrin, cypermethrin, etofenprox, and alpha-cypermethrin; see ‘Discussion’ section).

*Dengue incidence* - The history of dengue cases was scrutinized using the SINAN NET database (a Brazilian Health Information System).^50^ According to the PNCD, all confirmed dengue cases have been recorded in this database. Human population growth over the period examined was estimated based on the results of the censuses of the years 2000 and 2010, which were obtained at the website of the Brazilian Institute of Geography and Statistics (IBGE).^51^ These values were used to calculate the dengue incidence between 2000 and 2012. The dengue incidence was calculated as: (N/P) × 100,000 inhabitants, where N is the number of confirmed new dengue cases and P is the total resident population in a given time period.^52^ According to the MoH, values of dengue incidence above 300 cases per 100,000 inhabitants (or 0.3%) are considered epidemic.^3^

**Fig. 1:**
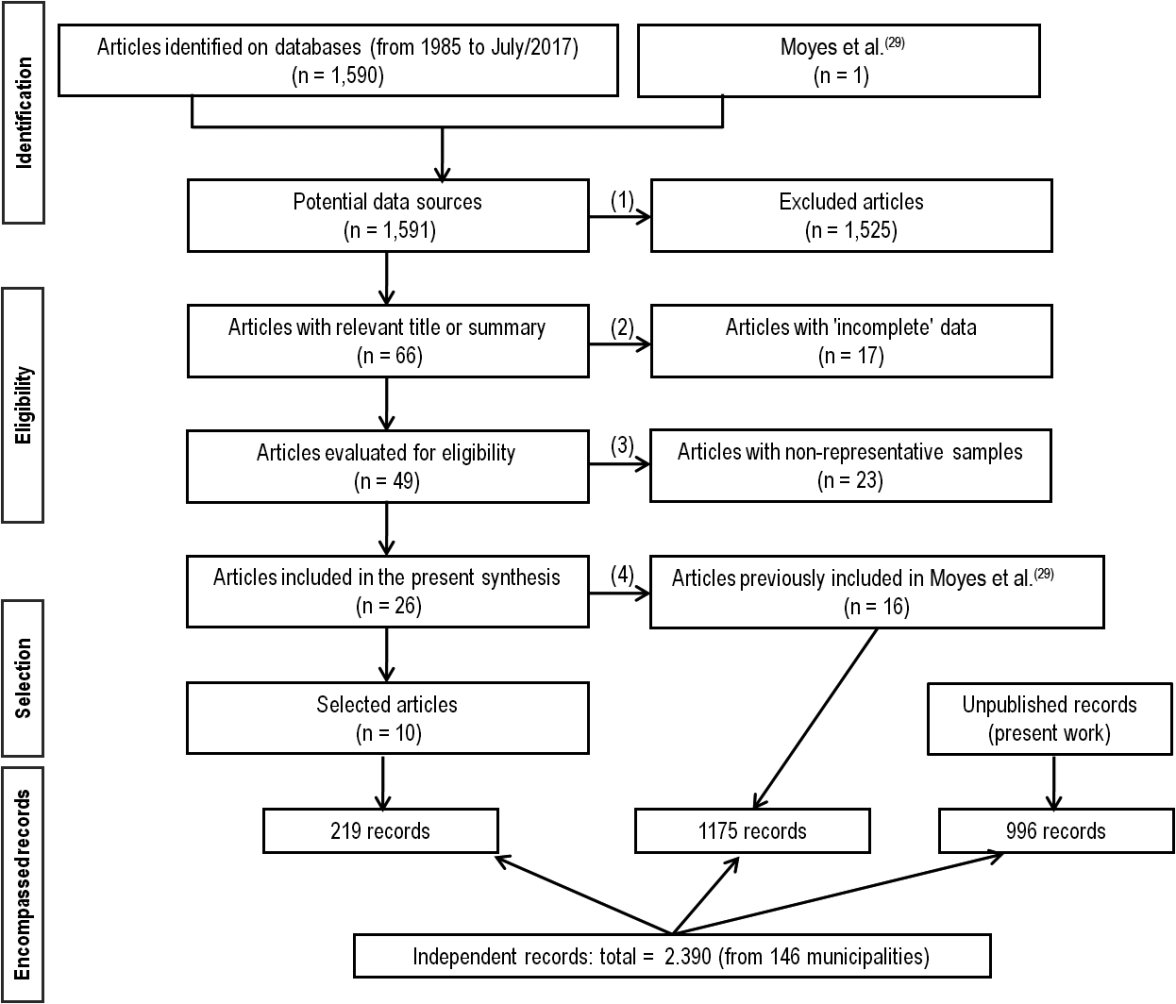
preferred reporting items for systematic reviews and meta-analyses (PRISMA) diagram, showing the different phases of the bibliographic survey and consolidation of the data regarding the insecticide resistance of Brazilian *Aedes aegypti* populations between 1985 and 2017. The following criteria were adopted for the inclusion of articles in the systematic review: (1) articles with a title and abstract related to the theme of this study were included. (2) Consistency of susceptibility status data against temephos or deltamethrin: for qualitative trials, those studies reporting the diagnostic concentration(s) of pesticide tested and the percent mortality obtained were included; for quantitative trials, those reporting LCs (50, 90, and 95) and the corresponding RRs were included. (3) Field collection: articles were included that provided information on the field sample size collected, taking into account their representativeness in relation to the municipality area and the collection period. For samples collected in districts or in other municipality subdivisions, the average RR of the reported values was considered; in these cases, the entire range of LC confidence intervals was used. Papers that did not mention the year of sample collection were discarded, except for those described by Braga et al.^(54)^, whose records are in our team’s possession. (4) The articles already covered by Moyes et al.^(29)^ were excluded from the present analysis.

*Bibliographical research and criteria for data inclusion in the survey* - An update on the temephos and deltamethrin resistance status of Brazilian *Ae. aegypti* populations from 146 municipalities is presented herein. The data included in the survey consisted of: (1) unpublished data generated by our team within the MoReNAa; (2) data from the recent Moyes et al.^29^ review, which encompassed populations from around the world, and updated the work of Ranson et al.^28^; (3) a systematic bibliographic survey of data from 1 January 1985 to 31 July 2017 in the databases contained in the National Center for Biotechnology Information (NCBI) (PubMed and PubMed Central ® - PMC) and in Google Scholar. In this survey the following search terms were used: ‘Brazil’, ‘*Aedes aegypti*’, ‘insecticide resistance’, ‘organophosphate’, and ‘pyrethroid’. Study selection was initially based on the relevance of the title and compatibility of the abstract with the theme of this study. The chosen articles were then read in full and submitted to a new sorting stage, this time according to other criteria: (1) the sample representativeness of each municipality, and (2) the presence of data on temephos and deltamethrin susceptibility status. Only articles describing representative field collections were kept (in some cases, the origin of the samples was not even reported). Fig. 1 shows the information flow in this systematic bibliographic review, and the quantity of unpublished records presented in this study; results were assembled according to the preferred reporting items for systematic reviews and meta-analyses (PRISMA) methodology proposed by Moher et al.^53^

*Ethics statement* - The use of anesthetised guinea pig to blood feed mosquitoes was authorised by the Fiocruz Ethical Committee for Animal Use (CEUA/Fiocruz), under license numbers P-0147/02, L-011/09, and LW-20/14.

## RESULTS

**Fig. 2:**
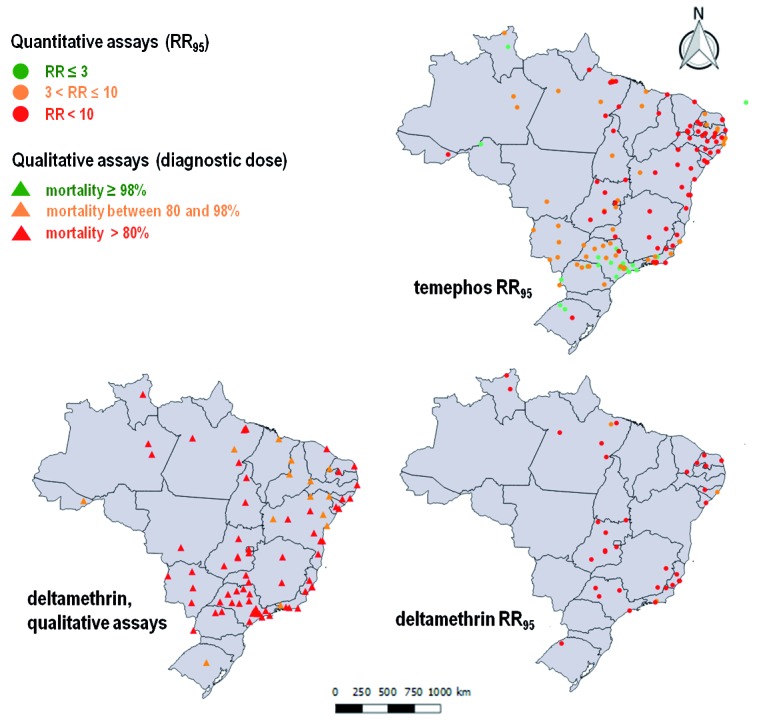
resistance status of larvae and adults of Brazilian *Aedes aegypti* populations against temephos and deltamethrin, respectively. For each municipality, the results of the most recent bioassay are shown. For deltamethrin, in addition to the results of quantitative (circles) bioassays, those of qualitative assays (triangles) are also depicted.

This study presented the results of evaluations of 146 Brazilian municipalities widely dispersed throughout the country, conducted between 1998 and 2012. Fig. 2 depicts the most current results of the 457 evaluations for temephos and 274 evaluations for deltamethrin, comprising the resistance status of Brazilian *Ae. aegypti* populations from 514 combinations of municipalities and years (‘municipalities/years’) and totalling 2,390 records. For each ‘municipality/year’, the total number of records was variable, and depended on both the insecticides evaluated and the tests performed (qualitative/quantitative). One ‘register’ was defined for each test: (1) in the case of qualitative assays, this was the mean mortality value; (2) for dose-response tests, this was each available LC (50, 80, 90, and 95) and the corresponding RR; and (3) for biochemical assays, this was the activity quantified for each enzyme class (ACE, MFO, GST, α-EST, β-EST, and ρNPA-EST). Allelic frequencies of *kdr* at the *Na*_*V*_ 1016 and 1534 positions, which are strongly associated with PY resistance,^55^ ^,^ ^56^ were also reported whenever available, although these were not considered as main ‘registers’ herein.

Data were presented separately for each geographical Region of Brazil, and organized sequentially by state and then municipality. For each municipality, the annual results were grouped chronologically. Supplementary data (Tables) are included that depict: the annual incidence of dengue in each municipality in the period evaluated [Supplementary data (Table II)]; the history of insecticide distribution by the MoH to each state for use against larvae [Supplementary data (Table III)] and adults [Supplementary data (Table V)]; results of bioassays of larvae exposed to temephos [Supplementary data (Table IV), quantitative assays]; and results of adult bioassays against deltamethrin [Supplementary data (Tables VI-VII) for qualitative and quantitative assays, respectively]. Supplementary data (Table VIII) exhibits the results obtained concerning the resistance status and mechanisms for all samples for which biochemical or molecular data were available.

In the text, in addition to sections dealing with each geographic region, two further sections were included: the first compared the results of CDC and WHO qualitative bioassays with adults, and the second analysed the relative participation of metabolic and target-site mechanisms in the resistance observed.

Our aim was to present, as completely as possible, the historical sequence of the development of resistance in Brazilian *Ae. aegypti* populations to insecticides used by public managers. It is noteworthy that 96.8% of these records were generated in laboratories belonging to the MoReNAa.

North Region

*Dengue incidence* - Most of the evaluated municipalities in this region were concentrated in Pará state, where dengue outbreaks occurred with a high, but sporadic, incidence [Supplementary data (Table II)]. However, some epidemics were reported in other states, even in consecutive years, and sometimes with incidences exceeding the ‘threshold’ of 0.3% by 5-30 times, including: Rio Branco, AC (2009-2011), Oiapoque, AP (2007-2011), and Boa Vista, RR (between 2001-2003 and 2008-2010). In Pacaraima, RR and Palmas, TO, high dengue incidence values were registered during most of the evaluated period.

*Resistance against the larvicide temephos* - During the evaluated period, temephos was almost continuously supplied throughout the North Region. In general, other products, such as *Bti* or IGRs, did not replace this larvicide, but were sometimes employed together with it [Supplementary data (Table III)]. Accordingly, resistance to temephos was found to be widespread in nearly all 20 evaluated municipalities in the North Region. The only exceptions were Porto Velho, RO in 2002^54^ and Boa Vista in 2010 [Supplementary data (Table IV)].

For the most part, the temephos RR_95_ values of mosquitoes in the North Region ranged from 4-12 in the studied period. The main exception was Oiapoque in 2009, in which very high resistance levels, including a RR_50_ of 42.1 and RR_95_ of 102.5, were recorded.^57^ This latter value was five times higher than the second largest RR_95_ in the region across the whole studied period: 21.4, in Dom Eliseu, PA in 2003.^20^ More recent trials with samples from Amapá (collected between December 2014 and January 2015) showed a significant reduction in temephos resistance, including a RR_50_ of 21.8 for mosquitoes from Oiapoque and of 6.5 for those from Macapá.^58^ It is worth remembering that temephos has been gradually replaced by IGRs since 2010, and its supplying to Amapá stopped in 2013 [Supplementary data (Table III)].

Oiapoque is located along the Brazilian border with French Guiana. The high temephos RR in Oiapoque in 2009 (RR_95_ = 102.5) may have been the consequence of the intense vector control actions applied in this border area by both countries. Dengue incidence was roughly 10 times higher than the threshold that defines epidemics in 2007-2011. This profile, however, does not seem to be general, as different patterns occurred in: (1) Palmas, TO, which presented RR values lower than 10 in two evaluations (2006 and 2009), with a high incidence of dengue in all years between sample collections; and (2) Rio Branco, which had a RR value below 10 in 2011, after four successive years of high incidences of dengue, with rates that reached 30 times the epidemic threshold. Therefore, it is not simply the epidemic *per se* that is associated with resistance, but the kind of response enacted to it, in particular the relative importance attached to chemical control of vectors.

*Resistance to deltamethrin* - In the North Region, deltamethrin was widely used in the control of adult mosquitoes over the period examined. Malathion was supplied to Pará in 2011, and later began to be employed in some other states [Supplementary data (Table V)]. Qualitative bioassays with deltamethrin using the WHO methodology classified 15 of the 18 evaluated mosquito populations as resistant, and none were considered susceptible.^43^ On the other hand, trials with the CDC methodology resulted in higher mortalities, and of the 11 evaluated populations, only two were classified as resistant [Supplementary data (Table VI)]. A comparison of these two methodologies is presented below (section: ‘Qualitative bioassays with adults: CDC and WHO methodologies’).

Although performed with mosquitoes from municipalities distinct from the sources of those used in qualitative tests, the results obtained with quantitative assays corroborated the changes in deltamethrin susceptibility suggested by the results of the former trials [Supplementary data (Tables VI-VII)]. The deltamethrin RR_95_, a parameter evaluated since 2009, was always high, usually between 8.5 and 26.6. However, for mosquitoes from Pacaraima, RR and Marabá, PA evaluated in 2011, the deltamethrin RR_95_ was extremely high, 60.3 and 70.7, respectively. Pacaraima borders Santa Helena, Venezuela, where there is more intense chemical control of malaria vectors; this and the high dengue fever incidence there since 2007 [Supplementary data (Table II)] may explain the elevated deltamethrin RR_95_ in mosquitoes from Pacaraima. On the other hand, in Marabá, despite the low incidence of dengue [Supplementary data (Table II)], there was an eight-fold increase in the RR_95_ in a two-year interval, from 8.8 in 2009 to 70.7 in 2011 [Supplementary data (Table VII)]. This was the highest deltamethrin resistance ratio found in the region. Altogether, for the North Region it was not possible to make the general conclusion that high dengue incidence corresponds with increased PY resistance. The increase in the use of PYs in general observed during epidemics due to blocking actions undertaken by public health services, intensification of domestic use, and non-integrated use of PYs against malaria vectors^10^ ^,^ ^13^ ^,^ ^14^ ^,^ ^59^ could explain the high level of resistance seen in mosquitoes from Pacaraima, but not in those from Marabá.

*Resistance mechanisms* - In the North Region, the activities of two enzymatic classes related to metabolic resistance were consistently altered: GSTs and, more intensely, ESTs, which in the latter case were evaluated with different substrates [Supplementary data (Table VIII)]. Few vector populations showed altered MFO activity. In only three of the 20 samples available, the total activity (AChE) of ACE, the target of OPs, appeared to be increased. However, in all cases ACE was considered sensitive to inhibition by insecticides according to WHO criteria (data not shown). Unfortunately, the metabolic resistance of larvae from a same locality in the North Region was never evaluated at two consecutive periods. However, there were simultaneous evaluations of larvae and adults on six occasions, and in all these cases the activity was more strongly altered in adults [Supplementary data (Table VIII)].

Northeast Region

*Dengue incidence* - Dengue dynamics between 2001 and 2012 in the Northeast Region presented four three-year periods with distinct disease incidences [Supplementary data (Table II)]; in the first and last of these, 2001-2003 and 2010-2012, dengue incidences were high and consistent with epidemic conditions in most of the evaluated locations in all states. In the second triennium (2004-2006), incidence values were the lowest observed, while in the third triennium (2007-2009) the dengue incidence was variable. It is noteworthy that this parameter was very high in Bahia during 2009. However, in the 2007-2009 triennium, the dengue incidence tended to be low in two periods and sections: in the beginning (2007), in the southernmost states of the Northeast Region (SE, BA); and in the last year (2009), in the remaining ones. In general, the number of ‘municipalities/years’ with a dengue incidence more than 0.3% corroborated this scenario: the first and fourth triennia registered higher numbers of 81 and 70 ‘municipalities/years’ that were above this cut-off point, respectively; whereas recorded values in the second triennium were lower (16), and in the third triennium they were intermediate (48).

Northeast Brazil was strongly affected by dengue over the evaluated period: in two states, CE and RN, at least 50% of ‘municipalities/years’ presented incidences indicating dengue epidemics. Even in MA, the least strongly affected state, more than 12% of the ‘municipalities/years’ presented high dengue incidences. The percentage of cases with incidences above 1,000 cases per 100,000 inhabitants, an arbitrary value equivalent to 1% of the population and more than three times the cut-off point defined by the Ministry of Health, was also evaluated. In this case, CE and RN were confirmed as the states that were the most strongly affected by dengue during the studied period. High incidences were also found for AL and BA.

*Temephos resistance* - Except for RN, temephos was continuously distributed to all states in the Northeast Region until 2011-2014 [Supplementary data (Table III)]. *Bti* was simultaneously applied in several states (MA, CE, PE, and AL) until 2009-2010; IGRs were also introduced during this period in most states in the Northeast Region. By 2014 almost all states exclusively employed IGRs.

In the Northeast Region, resistance to temephos was detected in all municipalities evaluated, with the only exception being Fernando de Noronha Island in 2009 [Supplementary data (Table IV)]. Although to date the highest temephos resistance levels in Brazil occur in the Northeast Region, the RR there varies significantly: a temephos RR_95_ above 100 was found in 20% of cases, but in almost 40% of cases the RR_95_ was below 10. The lowest dengue incidences and also the lowest temephos RR_95_ values (always below 10) were found in the state of MA. In contrast, extremely high temephos RR_95_ values above 100 (sometimes above 200) were concentrated in PE (six municipalities), southern CE (two), eastern BA (five), and the countryside of AL (one) [Supplementary data (Fig. 1)].

Much variation was noted in several aspects of insecticide resistance monitoring in the Northeast Region, including: (1) the number of municipalities evaluated in each state (ranging from only two in MA and PI, up to more than 10 in PE and BA); (2) the frequency of evaluation of the different municipalities, with each municipality evaluated only once in some states, such as MA and PB, while some municipalities in other states were evaluated five (Juazeiro do Norte, CE and Natal, RN), six (Fortaleza, CE and Maceió, AL), or even seven (Aracaju, SE) times over the evaluated period; and (3) the regularity of monitoring, as for example on the one hand five of the six evaluations were performed in 2003 for PB, while on the other hand in BA only three out of 23 evaluations were done before 2008.

For municipalities that were evaluated more than once, temephos resistance levels were compared with both their dengue incidence and supply of insecticides [Supplementary data (Tables II, III, IV). Although several different parameters influence dengue dynamics, some correlations were suggested, as outlined below:

― Crato, in the south of CE state, where the temephos RR_95_ in 2009 was higher than 190, presented a history of severe dengue epidemics between 2001 and 2007, which were five years with high values of dengue incidence, including two years when this was greater than 1%. This scenario may have contributed to the intensification of chemical control and the consequent increase in temephos resistance levels in this municipality. However, although dengue incidence remained high in this municipality until at least 2012, in 2013 resistance to temephos had declined more than three-fold (RR_95_ = 65), probably due to the interruption of its supply to the state in 2011.

― In Fortaleza, CE, the temephos RR_95_ increased from 8-10 in 2002 and 2006 to 43 in 2007. The annual dengue incidence was above the MoH cut-off point five times between 2001 and 2007, pointing to the intensification of chemical control and a consequent RR increase. Unfortunately, although there were several periods of high dengue incidence until 2012 in Fortaleza, no further temephos evaluations were reported there.

― With the exception of Santana do Ipanema, in the state of AL relatively stable temephos RR_50-95_ values, varying between four and 17, were found. These were consistent data, since these municipalities were assessed three to six times during the studied period. These results are also coherent with the supply of larvicides provided to AL state, since temephos was continuously but not exclusively provided between 2003 and 2013, being always concomitantly applied with *Bti* and/or an IGR.

― In contrast, in the state of RN, although temephos was provided only in 2004 [Supplementary data (Table II)], the RR_95_ of mosquitoes for this OP fluctuated over the same range as those in AL, and was around 12 (from 10 to 19) between 2004 and 2011. These are also consistent data, as of the five municipalities monitored, four were evaluated three to five times. This situation suggests that dengue incidence and records of insecticide supply by the MoH were not sufficient to explain the temephos resistance dynamics observed in RN state. Additional investigations could reveal other parameters that should be taken into account for the interpretation of these data.

― Accordingly, in Barra dos Coqueiros, SE, neither the low dengue incidence, nor the exclusive supplying of temephos as a larvicide over the evaluated period, were able to explain the increase in the temephos RR by seven times between 2000 (3.2) and 2004 (23.5).

In BA state, temephos was supplied continuously between 2003 and 2013, and this was the only larvicide used between 2004 and 2009. Among Bahia municipalities monitored more than once, two distinct scenarios should be mentioned: (1) records of high dengue incidence and stable levels of temephos resistance were observed in Barreiras, which was subjected to two years of very high dengue incidence between 2008 and 2012 (up to 1.6%), although the temephos RR_95_ there remained around 5.0 throughout the evaluated period; and (2) records of high dengue incidence and increasing temephos resistance levels were noted in such municipalities as Itabuna, Jequié, and Jacobina. In Itabuna, the temephos RR_95_ increased from 18.6 in 2004 to 55.8 in 2013, and records of dengue incidence in this municipality were also extremely high, in the range of 3-7%, in 2009 and 2012. Jequié exhibited low dengue incidences between 2004 and 2008, but high values between 2009 and 2012, and this last period was concomitant with a three-fold increase in the temephos RR_95_ value of mosquitoes from there. In Jacobina, temephos resistance levels increased 10-fold in one year, between 2008 and 2009, when the RR_95_ reached 104. Four years later, the temephos RR_95_ more than doubled, reaching almost 230. Dengue incidence in this municipality was high, or very high, during almost the entire period evaluated (2001-2012).

*Resistance to deltamethrin* - The whole Northeast Region received an uninterrupted supply of PY insecticides between 2003 and 2014 [Supplementary data (Table V)]. Malathion was delivered to CE in 2006, and to BA in 2011. This OP started to be continuously distributed in addition to deltamethrin in CE in 2008, and in some other states in the Northeast Region as of 2010.

Mosquitoes from all of the 39 ‘municipalities/years’ from the Northeast Region evaluated for deltamethrin resistance with qualitative tests using the WHO methodology were classified as resistant (26) or with incipient resistance (13). Among the 14 samples evaluated with the CDC methodology, 11 were considered resistant (two) or with incipient resistance (nine). In 13 cases, discussed in a section below, the two methodologies were applied simultaneously [Supplementary data (Table VI)].

Quantitative tests were performed in eight municipalities from four states [Supplementary data (Table VII)], and confirmed that resistance to PY was prevalent throughout the Northeast Region. The RR_95_ values were always above 7.0 and, except for Mossoró in 2011 and Crato in 2013, remained below 20.

In the Northeast Region the deltamethrin resistance status was evaluated twice in two localities, Mossoró, RN and Aracaju, SE. In both cases, the deltamethrin RR_95_ increased and, accordingly, the frequency of *kdr* alleles at the PY target site also increased [Supplementary data (Table VIII)].

*Resistance mechanisms* - The activities of two classes of detoxifying enzymes, GSTs and ESTs, were altered by pesticides [Supplementary data (Table VIII)]. Both α-EST and β-EST activity were affected in larvae, while α-EST was the most strongly altered class in adults. Total ACE activity presented increased levels in only two cases. However, according to WHO criteria, ACE was sensitive to insecticide inhibition throughout the Northeast Region (data not shown). MFO and ρNPA-EST activities were altered in some cases, mainly in adults. In eight instances, larvae and adults were simultaneously evaluated, with greater enzyme activity alterations always observed in adults [Supplementary data (Table VIII)]. The two municipalities evaluated at more than one point in time, Aracaju and Caicó, exhibited a general increase in the activity of detoxifying enzymes in both larvae and adults.

Southeast Region

*Dengue incidence* - In the Southeast Region, SP state stands out, as detailed below. SP presented the longest insecticide resistance monitoring history and the lowest dengue incidence of all evaluated states; it is an example of the positive effects of both entomological surveillance and resistance management on dengue prevention. During the evaluated period (2001-2012), the dengue incidence was above 0.3%, the MoH cut-off point, in less than 20% of ‘municipalities/years’, and only 10% of cases presented a dengue incidence of more than 1% [Supplementary data (Table II)]. In contrast, ES was the state in the Southeast Region that was the most strongly affected by dengue in the studied period, as in more than 50% of the ‘municipalities/years’ in ES the dengue incidence was above the MoH recommendation, and 20% of these had a dengue incidence of more than 1%. RJ and MG states presented intermediate values: 45-55% and 15-20% of the ‘municipalities/years’ in these states exhibited dengue incidences above 0.3 and 1%, respectively.

*Temephos resistance* - In the Southeast Region in general, temephos was distributed continuously throughout the period evaluated. *Bti* was supplied simultaneously until 2007-2010, and thereafter IGRs were used [Supplementary data (Table III)]. RJ was an exception, since temephos and IGRs were provided intermittently from 2004 onward in this state, while IGRs began to be supplied continuously after 2008, before they were supplied to the other states in the Southeast Region.

Temephos resistance was evaluated in 42 municipalities, totalling 250 ‘municipalities/years’, in this Region. SP accounted for more than 80% of the records, among which 17 municipalities out of 20 were assessed several times, some almost annually and, in many cases, from 14 to 18 times over the 1998-2015 period. In contrast, in other states in the Southeast Region, the accomplishment of temephos bioassays, which started from 1999 (RJ) and 2005 (MG and ES) onward, extended only until 2011. In these states, most municipalities were evaluated once or twice in the studied period, with a maximum of five evaluations in the case of Rio de Janeiro city [Supplementary data (Table IV)].

This differential resistance monitoring and management effort was reflected in the susceptibility profiles obtained: the average temephos RR_95_ of mosquitoes from SP (3.5) was at least three times lower than that of those in the other states in the Southeast Region (RJ, MG, and ES: RR_95_ = 10.1, 11.4, and 13.3, respectively). In SP, the temephos RR_95_ was above 3.0 in 60% of cases, in contrast with it being above this value for 100% of the municipalities in the other states, with the only exception to this trend being São José do Vale do Rio Preto, RJ (RR_95_ = 3.0). In SP, the temephos RR_95_ was above 10.0 in mosquitoes from only 1% of ‘municipalities/years’ (two out of a total of 194 records). In contrast, more than 50% of the records for the other states in the Southeast Region reached this value. Out of the 42 municipalities assessed in the Southeast Region, four were classified as having mosquitoes susceptible to temephos (RR_95_ below 3.0), three of which were located in SP. In particular, just one among the four municipalities was evaluated more than once; this was Botucatu, SP, the mosquitoes from which remained susceptible to temephos during all six monitoring rounds carried out between 2004 and 2014. This municipality did not exhibit a dengue incidence above the epidemic ‘threshold’ over the entire evaluated period [Supplementary data (Table II)].

In SP, a temephos RR_95_ above 10.0 was detected only in mosquitoes from São Sebastião in 2004 and from Santos in 2005. In both municipalities, resistance ratio values decreased later on, culminating at levels indicating susceptibility in 2015. In SP, the highest temephos RR_95_ levels were reported in Santos, São Sebastião, São Paulo, and Itapevi. The first two of these were among the five SP cities evaluated herein with the highest dengue incidences in the period of 2001-2012.

On the other hand, in the Southeast Region, the ES state presented the highest dengue incidence and also the highest temephos RR_95_ in the evaluated period. These were consistent data, which were always derived from more than one evaluation. In the case of RJ, although most of the locations were evaluated more than once, there were gaps in the RR_95_ records, and in MG only the capital, Belo Horizonte, was evaluated more than once.

*Resistance to deltamethrin* - Supplementary data (Table V) shows the supply of adulticides provided by the MoH for all vector control programs, not just those targeting *Ae. aegypti* (see ‘Discussion’). PYs were widely distributed throughout the Southeast Region: SP received malathion during the whole period, and other states received it from 2007 onward, or later. It should be mentioned that SP stopped using PYs against the dengue vector in 2000, almost 10 years before their replacement in the rest of the country.^60^

Deltamethrin qualitative bioassays were performed for 115 and 75 ‘municipalities/years’ with the WHO and CDC methodologies, respectively [Supplementary data (Table VI)]. In all cases, established or incipient resistance was detected. Established resistance prevailed in 92% of the trials (n = 106) done with the WHO methodology and in 72% of the trials (n = 54) done with the CDC methodology. Simultaneous evaluations with the WHO and CDC methodologies were performed for 46 ‘municipalities/years’ (see section below in which both methodologies were compared). Accordingly, deltamethrin quantitative bioassays revealed high resistance levels in all cases [Supplementary data (Table VII)].

*Resistance mechanisms* - GSTs were the enzymes whose activities were the most strongly altered by pesticides in samples of larvae and adults from the Southeast Region [Supplementary data (Table VIII)]. In adults, the activity of EST enzymes, mainly a-EST and ρNPA-EST, were also altered. Changes in MFO activity were also identified in some samples. Increases in total ACE activity were detected in roughly 30% of the evaluated samples. However, ACE activity was never altered in both stages evaluated from the same ‘municipality/year’. In addition, the ACE activity in all samples was considered sensitive to insecticide inhibition (AChI) when the WHO criterion was used (data not shown). Whenever biochemical assays were available for both life stages (eight pairs of samples), metabolic resistance was higher in adults. Metabolic resistance was never evaluated twice in larvae from the same locality. However, for five municipalities, the metabolic resistance of adults was evaluated 2-3 times; despite this, there were no concurrent data on the application of quantitative bioassays or on *Na*_*V*_ allele frequencies, a situation that precludes the analysis of the main mechanisms involved in resistance in these cases.

South Region

*Dengue incidence* - Due in part to its milder climate, the South Region was the least strongly affected by dengue. Most municipalities in SC and RS states evaluated over the studied period did not register dengue epidemics. The exception was Ijuí, RS in 2010, where the dengue incidence reached 3.7%, more than 12 times the MoH cut-off point [Supplementary data (Table II)]. The state of PR was the most affected by dengue. In the South Region, a dengue outbreak first occurred in 2002 in Foz do Iguaçu, PR, which is located on the triple border of Brazil with Paraguay and Argentina. In 2003, there was an epidemic in three municipalities in northern central Paraná, Cambé, Ipiporã, and Londrina. From then on, new records of high dengue incidences only occurred in this state in 2007, and then again in 2010/2011. In the period 2007-2011, Foz do Iguaçu endured three years of extremely high dengue incidences 3-12 times the MoH threshold of 0.3%.

*Temephos resistance* - All states in the South Region received the larvicide temephos almost continuously between 2003 and 2014 [Supplementary data (Table III)]. RS also received *Bti* in 2006 and 2009. The supplying of IGR larvicides to PR occurred from 2010 onward. Both SC and RS received IGRs in 2014 for the first time.

The temephos resistance status was evaluated in 12 municipalities in the South Region, totalling 29 ‘municipalities/years’ [Supplementary data (Table IV)]. Foz do Iguaçu and Maringá, both in PR, were evaluated eight and six times, respectively, between 2001 and 2009. Among the remaining municipalities, six were evaluated only once, and four were evaluated 2-3 times. Although in 55% of the bioassays the RR_95_ was higher than 3.0, the MoH cut-off point, it was never higher than 7.0. Mosquitoes from Maringá in 2005 and Ibiporã in 2006 exhibited the highest resistance levels. In general, a slight increase in resistance to OP was noted in the South Region over time.

*Resistance to deltamethrin* - Pyrethroids were provided almost continuously and used nearly exclusively in the South Region [Supplementary data (Table V)]. PR also received the OP malathion from 2010 onward. Qualitative deltamethrin bioassays were performed for three PR locations. Eight ‘municipalities/years’ were evaluated by the WHO method, and four of these were also evaluated using the CDC methodology [Supplementary data (Table VI)], and susceptibility was not detected in any case. In the South Region, results of deltamethrin quantitative bioassays were only available for mosquitoes from Santa Rosa, RS, for which there was a RR_95_ of 33.3 in 2011 [Supplementary data (Table VII)].

*Resistance mechanisms* - In the South Region, resistance mechanisms were investigated only in mosquito larvae and adults from Santa Rosa, RS in 2011. The larval enzyme activity profile was unaltered, which was consistent with their susceptibility to temephos [Supplementary data (Table VIII)]. In adults that were resistant to deltamethrin, changes in EST activity were detected, particularly in the activities of α-EST and ρNPA-EST. The participation of *Na*_*V*_ alterations in resistance was also identified in this case. Despite showing an increase in their total ACE activity, mosquitoes from Santa Rosa were considered to be sensitive to insecticide inhibition according to the WHO criteria (data not shown).

Centre-West Region

*Dengue incidence* - All municipalities evaluated in this Region experienced at least one dengue epidemic year between 2001 and 2012 [Supplementary data (Table II)]. Brazil’s capital, Brasília, in the Federal District (DF), was the least strongly affected locality, with there being high dengue incidence only in 2010. In the MS municipalities evaluated, dengue incidences generally increased from 2006 onward and reached values up to 6.3%, more than 20 times the MoH threshold, as was seen in Campo Grande in 2007. In GO, the dengue incidence also reached very high values in several localities. In particular, high dengue incidences were observed in two neighbouring municipalities, Goiânia and Aparecida de Goiânia, throughout the period evaluated (2001-2012). In 2010, a strong dengue epidemic affected the entire Centre-West Region: in 16 of the 17 municipalities evaluated, the dengue incidence was above 0.3%, and in four this value was even above 3.0%.

*Temephos resistance* - Except in the DF, temephos was supplied continuously between 2003 and 2013 throughout the Region. In MS, *Bti* was used in addition to temephos between 2003 and 2009. IGRs were introduced from 2009-2010, becoming the sole larvicides provided by the MoH throughout the Centre-West Region in 2014 [Supplementary data (Table III)].

Mosquitoes from all municipalities in the Centre-West Region evaluated were resistant to temephos [Supplementary data (Table IV)], the only exception being those from Brasilia in 2008. Brasilia presented the highest number of evaluations, and also the lowest temephos resistance levels and the lowest dengue incidence rates, over the evaluated period. Only Cuiabá in 2005 was evaluated in MT state; its temephos RR_95_ was also among the lowest in the region, in line with the moderate dengue incidence rates recorded there up to this time.

The temephos RR_95_ in MS was always below 8.0 [Supplementary data (Table IV)], even in 2011, after a period of extremely high dengue incidence in 2006-2010 [Supplementary data (Table II)]. The simultaneous use of temephos and *Bti* [Supplementary data (Table III)] may have contributed to there being less selection pressure for OP resistance in this state.

In contrast, mosquitoes from GO showed the highest temephos resistance levels in the region, as 10 out of the 15 municipalities for which the RR_95_ was evaluated in this state had values that were above 10.0 [Supplementary data (Table IV)]. Notably, São Miguel do Araguaia, GO in 2012 presented the highest temephos resistance level in the Centre-West Region, even though 2012 corresponded to the second year of low dengue incidence after five consecutive years of high incidences in this locality. The contiguous municipalities of Goiânia and Aparecida de Goiânia are clear examples of how differences in chemical control management can have an impact on the development of resistance. Although dengue incidence was high in both municipalities during the entire evaluated period, temephos resistance levels were much lower in Goiânia than in Aparecida de Goiânia, suggesting there were distinct selection pressures in both localities. Indeed, in the routine insecticide resistance monitoring of Brazilian *Ae. aegypti* populations, some adjacent municipalities with different local dynamics of infestation control were purposely chosen for just this reason. This was done to emphasize to municipal public officials how much both surveillance and infestation control strategies can impact insecticide resistance and, consequently, influence the effectiveness of vector control.

*Resistance to deltamethrin* - Pyrethroid adulticides were distributed widely in the Centre-West region between 2003 and 2014 [Supplementary data (Table V)]. The introduction of malathion, in addition to deltamethrin, began in 2009.

All 20 sites submitted to qualitative trials using the WHO methodology [Supplementary data (Table VI)] were classified as having mosquitoes that were resistant (18) or with incipient resistance (two). Of these, 11 were also evaluated using the CDC methodology, and two were classified as susceptible, while the others were considered resistant (five) or with incipient resistance (four).

Results of deltamethrin quantitative bioassays were only available for municipalities in GO state since 2009, and the results mainly came from 2011 [Supplementary data (Table VII)]. In all cases, data on resistance levels were obtained one or two years after dengue outbreaks [Supplementary data (Tables II, VII). In particular, deltamethrin resistance levels in mosquitoes from Luziânia were so high that the RR_95_ could not be readily estimated from the adult specimens available, as their RR_80_ was already almost 170. It is remarkable that Luziânia was considered susceptible to this PY three years before this evaluation (2008) according to a qualitative bioassay done using the CDC methodology [Supplementary data (Table VI)]. Additionally, Luziânia was one of the municipalities in the Centre-West Region that was the least strongly affected by dengue epidemics in the studied period [Supplementary data (Table II)]. This high deltamethrin resistance level in Luziânia in 2011 can be explained if one takes into account that the samples used for these bioassays were collected during the periods with the highest dengue incidence in this locality, and precisely at the end of the epidemic period [Supplementary data (Fig. 2)]. Thus, the high resistance levels may have reflected the seasonality of infestations, as well as the intensification of the use of adulticides during epidemic periods. The participation of *kdr* mutations, which contribute to PY resistance, was also detected in mosquitoes from Luziânia; unfortunately, the absence of biochemical data precludes the conclusive evaluation of the main mechanisms involved in this resistance.

*Resistance mechanisms* - The activities of two enzyme classes, GSTs and ESTs, were greatly altered in both vector stages [Supplementary data (Table VIII)]. In some cases, there was also a high alteration of MFO activity, but mainly in adults. Although the total ACE activity was altered in mosquitoes from two populations, this enzyme was classified in all cases as being sensitive to insecticide inhibition according to the WHO criteria (data not shown). Larvae and adults from Aparecida de Goiânia, Goiânia and Rio Verde were evaluated more than once. In general, they showed increased alterations to the activities of their metabolic resistance enzymes over time. Metabolic resistance tended to be higher in adults in nine out of the 10 available pairs of simultaneous evaluations of both larvae and adults.

*Qualitative bioassays with adults: CDC and WHO methodologies* - In the routine evaluation of the resistance of adult Culicidae to neurotoxic insecticides, two qualitative bioassay methodologies are available and globally used, although there is only limited agreement between them.^45^ Originally, the CDC and WHO methodologies were designed to evaluate different parameters, being knockdown and mortality, respectively. However, the assessment of mosquito mortality with the CDC methodology after recovery from insecticide exposure for 24 hours has already been employed in previous studies.^23^ ^,^ ^24^ ^,^ ^29^ ^,^ ^36^ ^,^ ^40^ This approach is especially useful in evaluating resistance to pyrethroids, which have a strong knockdown effect. In practice, this evaluation procedure, when performed after 24 h of recovery, tends to increase the agreement between both methodologies, as stated elsewhere.^45^

In the present study, mosquitoes from 82 ‘municipalities/years’ were submitted to bioassays using both procedures, and the mortality results obtained from these tests after 24 h of exposure to deltamethrin were then compared. The WHO methodology classified 87.8% of the populations as resistant, 12.2% as presenting incipient resistance, and none as susceptible [Supplementary data (Table VI)]. Meanwhile, mortality levels obtained with the CDC methodology tended to be higher, resulting in only 43.9% of the same populations being classified as resistant, 45.1% as having incipient resistance, and 11% as susceptible.

We identified a weak positive correlation between the results of both methodologies (n = 82, R^2^ = 0.014, p < 0.001). However, regional differences were observed: a weak negative correlation between results was found for samples from the Southeast and South Regions, but the correlation was positive for samples from the remaining Regions. In particular, a strong and highly significant correlation was found for samples from the North Region (n = 8, R^2^ = 0.751, p < 0.002).

The Cohen’s kappa coefficient (κ) values obtained showed that there was reasonable agreement between the two methodologies’ results for the 82 evaluated pairs of samples (κ = 0.39084) when the criterion proposed by David and Zahar^43^ was employed. This criterion considers populations with mortality below 80% in these bioassays as being resistant. However, the agreement increased considerably (κ = 0.64995) when the most recent criterion proposed by the WHO,^46^ which uses 90% mortality as the cut-off point for resistance, was used instead. It is worth noting that all of the results used herein came from laboratories belonging to the MoReNAa, in which both methodologies were adopted following standardised procedures in the monitoring routine of *Ae. aegypti* adults for the country of Brazil.

Several particular aspects of the two methodologies, discussed in detail elsewhere,^45^ are thought to account for their differences in performance. Additionally, mortalities tend to be higher in the bottle assays used in the CDC methodology, wherein the mosquitoes are forced to make direct contact with the insecticide, since the entire inner surface of the bottle, including the cap, is impregnated with the product. In contrast, the WHO methodology includes non-insecticidal spots that can be used as refuges, specifically the screens at the ends of the cylindrical tubes. In principle, the CDC methodology is more versatile, since the user can impregnate their bottles relatively easily on their own, while the impregnation of papers depends on a more delicate process, or on their acquisition already impregnated with the product. However, there are limitations to the use of impregnated bottles, but not WHO tubes, in humid places, especially in the field, as there may be condensation under such conditions that may cause mosquitoes to adhere to the walls of the bottles.

*Resistance to pyrethroids: metabolic and target-site mechanisms* - Some studies have suggested that metabolic changes tend to result in lower levels of resistance to pyrethroids than changes in their target site, *Na*_*V*_ .^37^ ^,^ ^61^ ^,^ ^62^ To test this possibility, deltamethrin resistance ratios were compared with both metabolic resistance and *kdr* frequencies. Direct comparisons were also made between the two resistance mechanisms, metabolic and target-site. As stated in the ‘Materials and methods’ section, for each population the mean level of the changes in activity found for all enzymatic classes related to metabolic resistance was used as a metabolic resistance index. The sum of the allelic frequencies of *kdr* mutations at positions 1016 and 1534 was used to evaluate changes in the PY target site.

When all available data from the entire country were combined, the deltamethrin RR was directly proportional to the frequency of *kdr* mutations (n = 18, R^2^ = 0.301, p < 0.001) [Supplementary data (Fig. 3A)]. Therefore, the higher the resistance level, the greater the participation of target-site alterations was in the resistance observed. Simultaneously, the deltamethrin RR and frequency of metabolic resistance tended to be inversely proportional; in other words, higher PY resistance levels tended to be correlated with less participation by detoxifying enzymes in resistance (n = 24, R^2^ = 0.004, p = 0.288) [Supplementary data (Fig. 3B)]. However, the correlation in the latter case was much weaker and non-significant, which makes sense when one considers that one of the variables evaluated in this case is a secondary parameter, derived from the activity of several enzymatic classes with variable specificity.

The direct comparison between both resistance mechanisms, metabolic and target-site, confirmed that one mechanism or the other tended to participate in the development of resistance; in other words, the frequency of metabolic resistance seemed to be higher when the *kdr* frequency was lower (n = 19, R^2^ = 0.012, p = < 0.001) [Supplementary data (Fig. 3C)].

## DISCUSSION

Although in the ‘Results’ section the details of the data collected were presented separately for each Region, we also attempted to identify national patterns and trends in relation to each of the main topics addressed: (1) the dengue incidence in the period studied; (2) the supply of the main insecticides used in vector control by the MoH to each state; (3) temephos and deltamethrin resistance profiles; and (4) the main and potential resistance mechanisms involved.

*Dengue incidence* - Only the dengue incidence in localities monitored for insecticide resistance was presented herein. As mentioned above, 96.8% of these records were generated within the MoReNAa, which attempted to define ‘sentinel municipalities’ that were representative of the dengue epidemiology in their geographical areas. Hence, it was possible to sketch an approximate picture of the recent history of dengue outbreaks and vector control in different Brazilian Regions. Except for the South Region, which was affected by dengue epidemics later than the rest of the country, the dengue incidence in all other regions was higher than the MoH cut-off point of 0.3% in more than 30% of municipalities [Supplementary data (Table IX)]. In addition, except for the South Region, extremely high dengue incidences above 1% were observed in more than 10% of the cases examined. In particular, in the Centre-West Region almost 20% of the records were above 1% over the studied period.

Supplementary data (Tables X, XI) depict the dengue incidence in each year in each Region. In general, the high incidence of dengue fever recorded in 2001-2002 and from 2008 onward^63^ can be seen in these data. However, epidemics did not occur simultaneously in all regions of the country. In particular, it is worth noting (1) the late inclusion of the South Region in the area of epidemiological importance for dengue, and (2) the magnitude and persistence of dengue epidemics in the Centre-West Region, most notably between 2006 and 2010. Climate changes, reflected in changes in temperature and precipitation patterns over the evaluated period, together with distinct macro- and micro-scale social determinants, tended to favour the spread of the vector.^64^ However, the non-simultaneous transmission of dengue in the country may be the consequence of heterogeneous variations in such climatic changes among the different geographical regions, which would have had unequal effects on the biology of the vector therein.

*Provision of insecticides by the Brazilian Ministry of Health* - We used the annual MoH registry of the insecticides supplied to the different states aiming to control different arthropod vectors between 2003 and 2014. Although the available documents did not specify the target vectors for the different products, we sought to restrict the analysis to the insecticides known to be used against *Ae. aegypti*. However, in the case of adulticides, we opted to include all PY insecticides provided by the MoH, since there is partial overlap between *Ae. aegypti* occurrence and that of other vectors targeted by PYs that are relevant to public health. In addition, the common mode of action of PY insecticides, and consequently their potential to induce selection for the same resistance mechanisms, were considered.

Temephos was the most heavily used larvicide in the period of 2003-2014, when it was supplied almost continuously to nearly all Brazilian Regions. In fact, for a long time temephos was the only larvicide approved by the WHO for use in potable water containers. Since at least 2003, *Bti* has also been used throughout the country, usually in addition to temephos. Subsequently, IGRs were introduced, and their use as larvicides was established in almost the entire country from 2009-2010, except for the South Region, where these larvicides were introduced later. It is worth mentioning that since 2012^7^ Brazil has adopted a larvicide rotation approach to preserve the supply of the few available products in any given year. Chitin synthesis inhibitors, mainly diflubenzuron and novaluron, were the first IGRs to be employed in Brazil, and were later succeeded by juvenile hormone analogues, such as pyriproxyfen. In 2013, temephos ceased to be the first-choice larvicide for use in *Ae. aegypti* control, and since 2014 it has no longer been used, and has instead been replaced by IGR for use in larval control.^65^

According to the WHO, there are only a few options to use as adulticides, all of which are PY products except for the OP malathion.^27^ In the 2003-2014 period, deltamethrin was used practically throughout the whole country except in SP state, where due to resistance PYs have not been employed in the control of *Ae. aegypti* adults since 2000.^14^ ^,^ ^60^ Despite this, SP continued to receive PYs to control other arthropod vectors. Malathion started to be distributed in 2006-2008 to some states, mainly in the Northeast (CE in 2006) and Southeast (RJ in 2007 and MG in 2008) Regions. Later, due to the dissemination of high levels of PY resistance in *Ae. aegypti* populations throughout the country, malathion was gradually introduced instead for adult control,^25^ ^,^ ^26^ and its use was well-established in the country by around 2009-2011.

*Resistance to the larvicide temephos* - Since 2006, Brazil has considered *Ae. aegypti* populations with a RR_95_ to temephos of 3.0 or higher to be resistant to this larvicide. This change in classification, from 10.0 to 3.0, was based on functional and operational criteria. For functional validation, simulated field trials confirmed the reduction of the effectiveness of temephos in populations with RR_95_ values above 3.0.^20^ The operational criteria were related to the logistics of the insecticide substitution procedure in the country, which depends on coordination among the three public management levels (federal, state, and municipal), meaning that, in practice, it can take up to two years for a recommendation at the central level to be implemented in the field.

Today, temephos resistance is so widespread in Brazil that this OP is no longer considered as the first-choice larvicide for use against *Ae. aegypti*, and it has been replaced by other, non-neurotoxic products. Varied temephos resistance levels were observed in the country. In some cases, high temephos resistance levels occurred simultaneously with, or soon after, periods of elevated dengue incidence, suggesting the intensification of chemical control in response to outbreaks; in other cases, resistance levels were associated with the temephos supply, or its interruption. However, in many cases these indicators were not enough to explain the variety of situations observed. For instance, the South Region presented the lowest RR_95_ values throughout the period evaluated. This result is compatible with the late inclusion of the South in the ‘dengue scenario’. Low temephos resistance levels were also found in SP state, but in this case they were probably due to there being strong and coordinated monitoring and resistance management. The Northeast Region presented the greatest variation in temephos resistance levels, as well as the highest registered values, as the RR_95_ was above 100 in 20% of cases there, and in 6% of cases it was higher than 200 (five municipalities, three in PE and two in BA). According to Moyes et al.,^29^ Brazil is the country with the highest levels of resistance by mosquitoes to temephos in the world. In Martinique, the country with the second highest resistance levels on this scale, the highest recorded RR_50_ was 35 in Gros Morne in 2009.^66^ In general, these results revealed the influence of local public health actions on the establishment of temephos resistance and the fragility of general guidelines, which should contribute to the more rational use of chemical control and, ultimately, its preservation as a complementary alternative for vector control.

*Resistance to deltamethrin* - In Brazil, a few decades elapsed until the spread of temephos resistance reached levels that could compromise its use in *Ae. aegypti* control. However, in the case of PYs, which were introduced by the PNCD across the whole country in 2000, only a few years were enough to induce high resistance levels in populations of this vector to this class of pesticide.^10^ ^,^ ^13^ ^,^ ^23^ ^,^ ^67^

Compared with larvae, the evaluation of adult resistance is more labour intensive. It depends on the use of devices and consumable materials that are not always easy to obtain. In addition, since adult females are used instead of larvae, tests become more time-consuming and dependent on the availability of specimens. For these reasons, routine tests are generally qualitative ones, in which specimens are exposed to only one insecticide dose and samples are classified as resistant, susceptible, or with incipient resistance. Two main methodologies, referred to herein as the CDC and WHO methodologies, are available for such tests and use bottles or papers, respectively, which are impregnated with insecticide.^35^ ^,^ ^39^ The CDC methodology tends to result in higher mortality levels due to the nature of the assay itself. In Brazil, both methodologies have been employed to evaluate deltamethrin resistance. The WHO methodology did not identify any susceptible populations, and with the CDC approach only 8% of the samples were susceptible. Although both the CDC and WHO methodologies are effective to assess the susceptibility profile of adult mosquitoes, the variable correlation between the results of the two methods inhibits, and even precludes, the ability for comparisons to be made between their results. In this sense, we tend to agree with Owusu et al.,^45^ who suggested that dose-response trials should be adopted to produce more consistent evaluations.

In this sense, within the scope of the MoReNAa we started to introduce the use of quantitative deltamethrin assays using impregnated papers directed at adult specimens in 2009.^26^ ^,^ ^68^ To our knowledge this was the first quantitative evaluation of adults performed in the *Ae. aegypti* resistance monitoring routine. It was necessary to establish a series of conditions and parameters for this technique, as well as to define evaluation criteria. In the latter case, we adopted the cut-off established in Brazil in 2006 for temephos: that a RR_95_ above 3.0 is indicative of resistance.^19^

Quantitative trials with deltamethrin for 41 ‘municipalities/years’ were identified herein. Most tests (30) were performed in 2011-2012, and confirmed the dissemination of resistance of *Ae. aegypti* to PYs in Brazil. Except for the South Region, very high RR_95_ values around 50-70 were detected in all Regions, and were more frequent in municipalities in the Centre-West Region. The Northeast Region tended to house the vector populations with the lowest PY resistance levels. There have been reports in the literature that *Ae. aegypti* resistance to PYs is more strongly influenced by vector seasonality, and in particular by the seasonality of dengue epidemics, than larvicides.^10^ ^,^ ^13^ ^,^ ^67^ This is a clear manifestation of the conceptual confusion between ‘vector control’ and ‘chemical control of adult vectors’. Indeed, this is one of the hypotheses that explains the rapid spread of PY resistance throughout the world.^13^ ^,^ ^14^ ^,^ ^55^ ^,^ ^67^ ^,^ ^69^ ^,^ ^70^ ^,^ ^71^ ^,^ ^72^

When the introduction of DENV-4 was detected in Brazil during the course of a dengue outbreak in Boa Vista, RR in the North Region, a quick and significant increase in PY resistance was detected throughout the city, including in areas that had not received ultra-low volume applications from the public health sector.^10^ Additionally, in SP state, *Ae. aegypti* resistance to pyrethroid insecticides continued to persist in several localities, even 10 years after their discontinuation in the control of the dengue vector by SP state municipal health secretaries.^14^ In relation to the data presented herein, in several cases high deltamethrin resistance levels were preceded by dengue epidemics, confirming that intense and indiscriminate use of pyrethroids occurred during outbreaks. The scenario found in the Centre-West Region is quite illustrative of this point: in practically all municipalities evaluated in 2011/2012, the RR_95_ was around 50. Since 2006, this region has been subjected to dengue outbreaks, culminating in 2010, which was precisely when the average dengue incidence in the municipalities evaluated was approximately 1.7%, almost six times the MoH cut-off point. The case of Luziânia, GO is also particularly informative: in 2011, the highest dengue rates so far registered (above 1.4%), and also extremely high levels of deltamethrin resistance (RR_80_ = 167), were found, and a closer look at the data revealed that the collection of field material for these bioassays was performed at the end of the epidemic season [Supplementary data (Fig. 2)], showing the influence of the intensification of adulticide application on the vector populations’ pyrethroid resistance status.

*Resistance mechanisms* - Metabolic resistance is the result of changes in the activities of enzymes involved in the metabolism, sequestration, and excretion of insecticides. The activities of three major enzyme classes, MFOs, ESTs (Phase I), and GSTs (Phase II), were evaluated in larval and adult female samples using the routine monitoring methodology applied in Brazil. In general, adults exhibited greater alterations to their enzymes’ activities than larvae.

The multifactorial nature of insecticide resistance was confirmed in this study, as has been reported for other vector populations worldwide.^73^ ^,^ ^74^ ^,^ ^75^ The most strongly affected mechanisms of metabolic resistance throughout Brazil were the activities of the EST and GST enzyme superfamilies, which were closely related to OP and PY resistance in Brazilian samples.^13^ ^,^ ^20^ ^,^ ^57^ ^,^ ^67^ ^,^ ^76^ Changes in MFO activity were less prominent, as only around 30% and less than 20% of adult and larval samples, respectively, exhibited changes in MFO activity. This metabolic profile contrasts with those reported by studies done elsewhere,^56^ ^,^ ^70^ ^,^ ^77^ ^,^ ^78^ ^,^ ^79^ and this difference was probably due to the distinct nature of the assays examined herein. Specifically, these biochemical assays (which are adopted in routine monitoring programs) quantify levels of different enzyme classes, and thus may underestimate activity alterations; in contrast, the detection of MFO participation in metabolic resistance is generally derived from molecular assays that consist of more specific approaches to investigate gene expression changes. Efforts to understand the dynamics of metabolic resistance in Brazilian vector samples at the gene expression level, including those of MFOs, are currently underway.

The OP target is encoded by two genes, *ace1* and *ace2*.^80^ The relationship between some *ace1* mutations in mosquitoes belonging to the genera *Culex* and *Anopheles* and their OP resistance has been well-documented.^80^ ^,^ ^81^ The participation of two recently detected point mutations in *ace1* in OP resistance in *Ae. aegypti* populations in India and Indonesia has also been suggested.^82^ ^,^ ^83^ However, it is intriguing that for the mutations reported in *Aedes* mosquitoes, only a small difference in the inhibition profile of ACE has been suggested, which is different from the impacts of similar mutations observed in *Culex* and *Anopheles* species. The present study detected changes in total ACE activity (AChE) in 17% of the analysed samples, but in no case did the remaining ACE activity after insecticide inhibition (AChI) signal the alteration of this endpoint. However, it is important to note that the criteria applied to classify the ACE status in both assays, AChE and AChI, are quite different, which indicates the need for further, deeper analyses of this enzyme in *Ae. aegypti* field populations.

In relation to PY resistance, some research groups have argued that metabolic changes tend to result in lower resistance levels than alterations in the PY target site, *Na*_*V*_ .^37^ ^,^ ^61^ ^,^ ^62^ We presented evidence that the participation of metabolic mechanisms indeed seems to be limited in populations with high deltamethrin resistance levels. Accordingly, it has been shown elsewhere that there is less participation of the metabolic detoxification pathway in the resistance of mosquito populations presenting *Na*_*V*_ *kdr* mutations.^61^ In that same study, the authors suggested that different *Ae. aegypti* populations may respond to selection pressure in distinct ways, hindering or even preventing attempts to identify metabolic resistance components that could be diagnostic of PY resistance in general.

The history of temephos and deltamethrin resistance status examined herein provides evidence of the spread of resistance against the main insecticides used by the PNCD in the control of Brazilian *Ae. aegypti* populations. The high temephos resistance levels observed are the consequence of its use for more than 40 years in Brazil. Rapid dissemination of adult resistance to deltamethrin was correlated with the intensified chemical control of adults during dengue epidemic seasons. High dengue incidences are recurrent in Brazil, a hyperendemic country for this arbovirus. This dataset highlights the limitations of chemical vector control as the methodology of choice for the sustainable reduction of dengue incidence. In this sense, the rotation of insecticides has already been adopted in Brazil as a strategy to preserve these chemical pesticides as effective vector control tools. The multifactorial character of pesticide resistance was shown in this study. In view of the insecticide resistance of *Ae. aegypti* populations in Brazil, alternative approaches, such as community engagement and mechanical control, should be considered when planning interventions to reduce dengue transmission. It is worth emphasizing the importance of respecting the complementary character of insecticides, meaning that they should be reserved for the treatment of vector breeding places that cannot be eliminated and for specific places and situations, such as outbreak blockage or intervention at strategic points.
