# Supplementary material for: Resistance to temephos and deltamethrin in Aedes aegypti from Brazil between 1985 and 2017
Source: Mem Inst Oswaldo Cruz. 2019 Apr 29;114:e180544. doi: 10.1590/0074-02760180544 (PMC6489372; doi:10.1590/0074-02760180544)
Supplement: Supplementary file 1 [file 1678-8060-mioc-114-e180544-s.pdf]

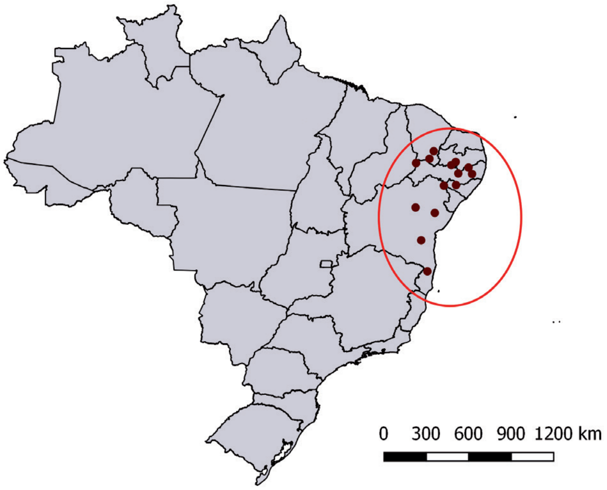

Fig. 1: Brazilian *Aedes aegypti* populations exhibiting unusually high resistance levels to temephos (i.e. RR_{95} above 100).

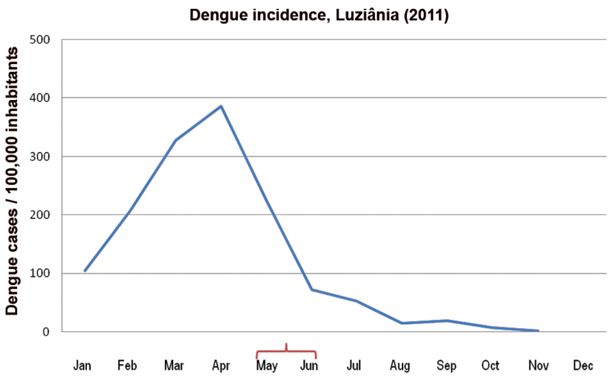

Fig. 2: incidence of dengue in the municipality of Luziânia, GO, in 2011. In red: collection period of *Aedes aegypti* eggs in the field. Bioassays with F1 specimens derived from this material resulted in a deltamethrin RR_{80} of 167.

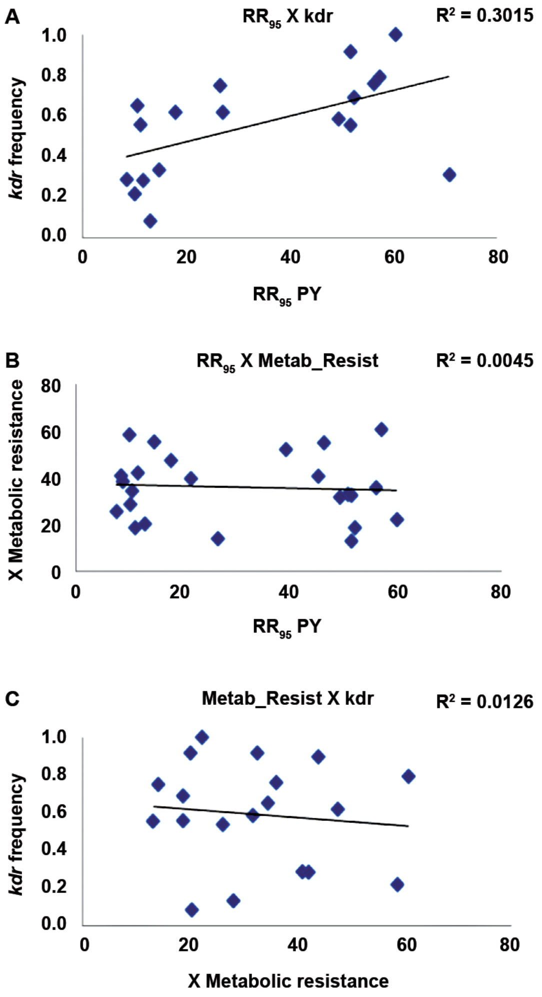

Fig. 3: resistance to pyrethroids (PY) and related mechanisms thereof. Comparisons between deltamethrin resistance levels (RR_{95} PY) and the two main mechanisms potentially involved are shown: (A) *kdr* mutations at the PY target site, (*Na_V*) and (B) metabolic resistance ('Metab_Resist'); (C) both mechanisms, target-site and metabolic resistance, were also directly compared.

SUPPLEMENTARY DATA (TABLES I - XII)

CC BY
